# Inhibition of in vivo proliferation of androgen-independent prostate cancers by an antagonist of growth hormone-releasing hormone.

**DOI:** 10.1038/bjc.1997.271

**Published:** 1997

**Authors:** A. Jungwirth, A. V. Schally, J. Pinski, G. Halmos, K. Groot, P. Armatis, M. Vadillo-Buenfil

**Affiliations:** Endocrine, Polypeptide and Cancer Institute, Veterans Affairs Medical Center and Department of Medicine, Tulane University School of Medicine, New Orleans, LA 70146, USA.

## Abstract

Tumour-inhibitory effects of a new antagonist of growth hormone-releasing hormone (GH-RH), MZ-4-71, were evaluated in nude mice bearing androgen-independent human prostate cancer cell lines DU-145 and PC-3 and in Copenhagen rats implanted with Dunning R-3327 AT-1 prostatic adenocarcinoma. After 6 weeks of therapy, the tumour volume in nude mice with DU-145 prostate cancers treated with 40 microg day(-1) MZ-4-71 was significantly decreased to 37 +/- 13 mm3 (P < 0.01) compared with controls that measured 194 +/- 35 mm3. A similar inhibition of tumour growth was obtained in nude mice bearing PC-3 cancers, in which the treatment with MZ-4-71 for 4 weeks diminished the tumour volume to 119 +/- 35 mm3 compared with 397 +/- 115 mm3 for control animals. Therapy with MZ-4-71 also significantly decreased weights of PC-3 and DU-145 tumours and increased tumour doubling time. Serum levels of GH and IGF-I were significantly decreased in animals treated with GH-RH antagonist. In PC-3 tumour tissue, the levels of IGF-I and IGF-II were reduced to non-detectable values after therapy with MZ-4-71. The growth of Dunning R-3327 AT-1 tumours in rats was also significantly inhibited after 3 weeks of treatment with 100 microg of MZ-4-71 day(-1) i.p. as shown by a reduction in tumour volume and weight (both P-values < 0.05). Specific high-affinity binding sites for IGF-I were found on the membranes of DU-145, PC-3 and Dunning R-3327 AT-1 tumours. Our results indicate that GH-RH antagonist MZ-4-71 suppresses growth of PC-3, DU-145 and Dunning AT-1 androgen-independent prostate cancers, through diminution of GH release and the resulting decrease in the secretion of hepatic IGF-I, or through mechanisms involving a lowering of tumour IGF-I levels and possibly an inhibition of tumour IGF-I and IGF-II production. GH-RH antagonists could be considered for further development for the therapy of prostate cancer, especially after the relapse.


British Journal of Cancer (1997) 75(11), 1585–1592
© 1997 Cancer Research Campaign

### Inhibition of in vivo proliferation of androgen-independent prostate cancers by an antagonist of growth hormone-releasing hormone

A Jungwirth*, AV Schally, J Pinski, G Halmos, K Groot, P Armatis and M Vadillo-Buenfil

Endocrine, Polypeptide and Cancer Institute, Veterans Affairs Medical Center and Department of Medicine, Tulane University School of Medicine, New Orleans, LA, USA

**Summary** Tumour-inhibitory effects of a new antagonist of growth hormone-releasing hormone (GH-RH), MZ-4-71, were evaluated in nude mice bearing androgen-independent human prostate cancer cell lines DU-145 and PC-3 and in Copenhagen rats implanted with Dunning R-3327 AT-1 prostatic adenocarcinoma. After 6 weeks of therapy, the tumour volume in nude mice with DU-145 prostate cancers treated with 40 µg $day^{-1}$ MZ-4-71 was significantly decreased to 37 ± 13 $mm^3$ ($P < 0.01$) compared with controls that measured 194 ± 35 $mm^3$. A similar inhibition of tumour growth was obtained in nude mice bearing PC-3 cancers, in which the treatment with MZ-4-71 for 4 weeks diminished the tumour volume to 119 ± 35 $mm^3$ compared with 397 ± 115 $mm^3$ for control animals. Therapy with MZ-4-71 also significantly decreased weights of PC-3 and DU-145 tumours and increased tumour doubling time. Serum levels of GH and IGF-I were significantly decreased in animals treated with GH-RH antagonist. In PC-3 tumour tissue, the levels of IGF-I and IGF-II were reduced to non-detectable values after therapy with MZ-4-71. The growth of Dunning R-3327 AT-1 tumours in rats was also significantly inhibited after 3 weeks of treatment with 100 µg of MZ-4-71 $day^{-1}$ i.p. as shown by a reduction in tumour volume and weight (both $P$-values < 0.05). Specific high-affinity binding sites for IGF-I were found on the membranes of DU-145, PC-3 and Dunning R-3327 AT-1 tumours. Our results indicate that GH-RH antagonist MZ-4-71 suppresses growth of PC-3, DU-145 and Dunning AT-I androgen-independent prostate cancers, through diminution of GH release and the resulting decrease in the secretion of hepatic IGF-I, or through mechanisms involving a lowering of tumour IGF-I levels and possibly an inhibition of tumour IGF-I and IGF-II production. GH-RH antagonists could be considered for further development for the therapy of prostate cancer, especially after the relapse.

**Keywords:** prostate cancer; PC-3; DU-145; Dunning tumour; growth hormone-releasing hormone antagonist; insulin-like growth factor

Carcinoma of the prostate is the most common malignant tumour in men. It is expected that in 1996 approximately 317 000 new cases of prostate cancer will be diagnosed in the US and that about 41 000 deaths will occur from this disease (Parker et al, 1996). In spite of refinements in surgical techniques (Walsh et al, 1994) and improvement of clinical outcome after radiotherapy (Garnick, 1993), many patients experience a recurrence. According to Lu-Yao et al (1996), 34.9% of patients studied after radical prostatectomy needed additional cancer therapy within 5 years of initial surgery. In addition to these highly selected patients with clinically localized prostate cancer at initial presentation, there is a large group of men with advanced stages of the disease at the time of primary diagnosis. Medical or surgical castration is the only established therapy for local recurrence or advanced prostate cancer (Schally et al, 1990) with an effective first-line response rate of 70–80% (Sharifi et al, 1990). However, all hormonal therapies aimed at androgen deprivation can only provide a remission, and most patients with advanced prostatic carcinoma will relapse in 18–36 months. The suppression of androgen-independent prostate cancer is a major oncological challenge, and new therapeutic approaches must be developed.

The PC-3 prostate cancer cell line was originally isolated from a metastasis of a human prostatic adenocarcinoma to bone (Kaighn et al, 1979). PC-3 cells show a reduced dependence upon serum for growth and cannot be stimulated by androgens (Kaighn et al, 1979). The DU-145 cell line, derived from a human prostate adenocarcinoma metastatic to the brain is also androgen independent (Stone et al, 1978). These two cell lines can be xenografted into nude mice. Dunning R 3327 AT-1, a subline derived from the rat Dunning prostate cancer, is an anaplastic, hormone-insensitive tumour, transplantable into rats (Isaacs et al, 1978). PC-3, DU-145 and Dunning R-3327 AT-1 cell lines are valuable models for investigating biological tumour behaviour and the effect of new anticancer drugs on androgen-independent prostate carcinoma.

The prostate is a hormone-sensitive organ, primarily under the control of the pituitary–gonadal axis (Schally et al, 1990; Reubi et al, 1995). However, some regulatory factors other than androgens have been identified, among them hormones, such as luteinizing hormone-releasing hormone (LH-RH), GH, somatostatin and bombesin-like peptides and growth factors, including epidermal growth factor (EGF), transforming growth factors-α and -β (TGF-α, TGF-β), IGF-I and IGF-II (Davies and Eaton, 1991; Schally et al, 1990; Reubi et al, 1995; Angelloz-Nicoud and Binoux, 1995;

*Received 24 September 1996*
*Revised 27 November 1996*
*Accepted 4 December 1996*

*Correspondence to:* AV Schally, VA Medical Center, 1601 Perdido Street, New Orleans, LA 70146, USA

*On leave from the Department of Urology, Salzburg General Hospital, Salzburg, Austria

Reiter et al, 1995). These substances can directly or indirectly regulate the function and growth of the prostate, especially after malignant transformation. Synthesis of IGF-I is induced primarily by GH but, in addition, growth factors like EGF and proto-oncogenes like c-*myb* may be involved (Pietrzkowski et al, 1993). IGF-I is synthesized and secreted mostly by the liver, but also by other tissues, such as lung and kidney (Froesch et al, 1985). Various cancer cells are likewise able to produce and secrete insulin-like growth factors (Macaulay, 1992), among them PC-3 and DU-145 prostate cancer cell lines (Pietrzkowski et al, 1993). Type I IGF receptors (IGFRs) have been shown on membranes of DU-145 and PC-3 cells (Pietrzkowski et al, 1993). Furthermore, there is some evidence that IGF might be responsible for the progression of prostate cancer in the advanced stages. Consequently, the blocking of the GH–IGF axis might lead to improvement in the treatment of advanced, androgen-independent prostate cancer.

Recently, various potent GH-RH antagonists have been synthesized in our laboratory, including [$Ibu^0$, D-$Arg^2$, Phe(4-Cl)$^6$, $Abu^{15}$, $Nle^{27}$] hGH-RH(1–28)Agm (MZ-4-71) (Zarandi et al, 1994). This antagonist has previously been shown to inhibit growth of human osteosarcomas (Pinski et al, 1995) and human small- and non-small-cell lung cancers in athymic nude mice (Pinski et al, 1996). In the present study, we investigated the effect of the GH-RH antagonist MZ-4-71 on the growth of the hormone-independent prostate cancer cell lines DU-145 and PC-3 xenografted into nude mice, and rat Dunning R-3327 AT-1 tumours transplanted into Copenhagen rats. In addition, we examined the effect of this antagonist on the proliferation of DU-145 and PC-3 cells in vitro.

### MATERIALS AND METHODS

### Peptide and reagents

The GH-RH antagonist MZ-4-71, ([$Ibu^0$, D-$Arg^2$, Phe(4-Cl)$^6$, $Abu^{15}$, $Nle^{27}$] hGH-RH(1–28)Agm) was synthesized by solid-phase methods and purified in our laboratory (Zarandi et al, 1994). For daily injections, MZ-4-71 was dissolved in 0.1% dimethyl sulphoxide (DMSO) in 10% propylene-glycol in saline solution.

### Animals

Male athymic (NCr *nu/nu*) nude mice, approximately 6 weeks old on arrival, were obtained from the National Cancer Institute (Bethesda, MD, USA) and housed in laminar airflow cabinets under pathogen-free conditions with a 12-h light/12-h dark schedule and fed autoclaved standard chow and water ad libitum. Their care was in accord with institutional guidelines. Copenhagen male rats were obtained from Charles River Laboratories (Frederick, MD, USA). They were housed four to a cage in a temperature-controlled room with a 12-h light/12-h dark schedule and fed water and standard rat chow ad libitum.

### Cell culture

The human androgen-independent prostatic carcinoma lines DU-145 and PC-3 were obtained from the American Type Culture Collection (ATCC), Rockville, MD, USA. DU-145 and PC-3 cells were grown as a monolayer in RPMI-1640 medium (Gibco, Grand Island, NY, USA) supplemented with 10% and 5% fetal bovine serum, respectively, antibiotics and antimycotics, at 37°C in a humidified 95% air / 5% carbon dioxide atmosphere. Tumour cells growing exponentially were harvested by brief incubation with 0.25% trypsin-EDTA solution (Gibco). Xenografts of PC-3 and DU-145 were initiated by subcutaneous (s.c.) injection of $1 \times 10^7$ cells into the left flanks of five male nude mice for each tumour cell line.

### Experimental protocol

#### *Experiments I and II*

PC-3 and DU-145 tumours resulting after 8 weeks of growth were aseptically dissected and mechanically minced; 3-$mm^3$ pieces of each tumour tissue were transplanted s.c. by trocar needle into two groups of 30 male animals under methoxyflurane (Metofane; Pitman-Moore, Mundelein, IL, USA) anaesthesia. The tumour take rate was 70% for DU-145 and 90% for PC-3 tumours. Three weeks after transplantation, DU-145 or PC-3 tumours had grown to a volume between 17 and 24 $mm^3$. Mice bearing PC-3 or DU-145 tumours were then divided in both experiments into two groups of eight animals each. Control groups were injected only with 0.1% DMSO in 10% propylene-glycol/saline, s.c.; and experimental groups were treated with MZ-4-71, 20 μg twice a day, s.c. The treatment was continued for 6 weeks for DU-145 tumours and for 4 weeks for PC-3 tumour-bearing nude mice. The tumours were measured once a week with microcalipers, and the tumour volume was calculated as length × width × height × 0.5236 (Janik et al, 1975). Tumour volume doubling time was calculated between the start and the end of the experiment. At the end of the treatment period, mice were anaesthetized with methoxyflurane, killed by decapitation and trunk blood was collected. The serum was separated and frozen for further analyses. Body weights were recorded and various organs were removed and weighed. Tumours were carefully cleaned and weighed, and samples were taken for receptor studies. Tumour samples and liver tissues were collected for measurements of tissue levels of IGF-I and -II.

#### *Experiment III*

Dunning R-3327 AT-1 tumour-bearing rats were kindly provided by Dr John T Isaacs (The Johns Hopkins Oncology Center, Baltimore, MD, USA). The tumour was aseptically dissected and mechanically minced; 3-$mm^3$ pieces of tumour tissue were transplanted s.c. in the scapular region by trocar needle into five male Copenhagen rats under methoxyflurane anaesthesia. Two weeks after transplantation, tissue obtained from growing tumours was transplanted subcutaneously into 20 male Copenhagen rats under methoxyflurane anaesthesia. Treatment was started immediately after tumour transplantation. The rats were divided into two groups (ten rats per group): group 1 (control) received 0.1% DMSO in 10% propylene-glycol/saline, i.p.; group 2 was injected with 100 μg of MZ-4-71 per animal per day i.p., for 3 weeks. All experiments were approved by the institutional ACUC and the procedures were essentially in accordance with UKCCCR guidelines for the welfare of animals in experimental neoplasia.

### Method of tissue extraction

Tumour and liver tissue concentrations of IGF-I and IGF-II were determined by an adaptation of the methods described previously (D'Ercole et al, 1984). The tissue was cut in small slices and homogenized in 5 ml of 1 M acetic acid 1 $g^{-1}$ tissue using Ultra Turrax homogenizer at 4°C. The homogenate was centrifuged at

 *© Cancer Research Campaign 1997*

2000 × *g* for 20 min at 4°C. The supernatant was collected and the pellet was washed and recentrifuged. The supernatants were combined, lyophilized and reconstituted in 0.1 M phosphate buffer. The BIO-RAD protein assay kit (Hercules, CA, USA) was used for protein determination.

### Radioimmunoassays of GH, IGF-I and IGF-II

Serum GH was determined using materials provided by Dr AF Parlow (Pituitary Hormones and Antisera Center, Torrance, CA, USA; mouse GH reference preparation AFP10783B, mouse GH antigen AFP10783B and anti-rat GH-RIA-5/AFP-411S). All serum and reconstituted tissue samples for IGF-I and IGF-II determination were extracted by a modified acid–ethanol cryoprecipitation method described previously (Daughaday et al, 1980; Breier et al, 1991). This method eliminates most of the IGF binding proteins, which can interfere in the radioimmunoassay (RIA). The extracted IGF-I was measured by RIA using IGF-I (88-G4, from Genentech, San Francisco, CA, USA) for standard in the range of 2–500 pg per tube and for iodination using the standard chloramine-T method. Antibody UB3-189 and UB2-495 (a gift from Dr Underwood and J van Wyk) obtained from NIDDK was used at the final dilution of 1:10 000 and 1:14 000 in the RIA.

IGF-II was measured using human recombinant IGF-II (Bachem California, Torrance, CA, USA) in the range of 2–500 pg per tube. IGF-II was iodinated by lactoperoxidase method and purified by reverse-phase high-performance liquid chromatography (HPLC) using a Vydac C18 column. For the assay, Amano monoclonal antibody generated against rat IGF-II (10 μg $ml^{-1}$) was used at a final dilution of 1:14 285 (Amano Enzymes USA, Troy, VA, USA). This antibody cross-reacts 100% with human and rat IGF-II and 10% with human IGF-I (Tanaka et al, 1989).

### Receptor assay

Measurement of receptors for IGF-I in the membranes of DU-145, PC-3 and Dunning R-3327 AT-1 tumours was performed as described previously (Pinski et al, 1995). The LIGAND PC computerized curve-fitting program of Munson and Rodbard (1980) was used to determine the types of receptor binding, dissociation constant ($K_d$) values and the maximal binding capacity ($B_{max}$) of receptors.

### Growth in serum-free medium

For the MTT assay, cells were seeded into 96-well microplates (Falcon, Lincoln Park, NJ, USA) in serum-free medium. The medium contained RPMI-1640 (Gibco), 0.5% bovine serum albumin (BSA), 1 mM pyruvate and 1 μM ferrous sulphate (all from Sigma).

### MTT assay

This assay is based on a method described by Plumb et al (1989). Briefly, cells were seeded into 96-well microplates and cultured for 18 h. MZ-4-71 was added to the medium in final concentrations of $10^{-7}$–$10^{-5}$ M. Control cultures received serum-free medium alone. After 72 h of culture, the medium was removed and 200 μl of serum-free medium containing 80 μg of MTT [3,(4,5-dimethylthiazol-2yl)-2,5-diphenyl tetrazolium bromide; Sigma] was added. The microplates were incubated for 4 h at 37°C in darkness. The medium was removed, cells were washed with RPMI-1640 medium, then 200 μl of dimethyl sulphoxide (DMSO; Sigma) was added, followed by 25 μl of Sorensen's glycine buffer (0.1 M glycine plus 0.1 M sodium chloride, pH 10.5). After a brief mixing, the plates were read at 540 nm on the plate reader (Beckman, Palo Alto, CA, USA). Results were calculated as % *T/C*, where $T$ = optical density ($OD_{540nm}$) of treated cultures (serum-free medium plus MZ-4-71) and $C$ = $OD_{540nm}$ of control cultures (serum-free medium alone) × 100.

### Statistical methods

All data are expressed as means ± s.e.m., and statistical analyses of the tumour data were performed using Duncan's new multiple range test (Steel and Torrie, 1976). All *P*-values are based on two-sided hypothesis testing.

### RESULTS

### Effects of GH-RH antagonist MZ-4-71 on growth of DU-145 and PC-3 tumours in nude mice

At the end of the experiments, there were no significant differences in body weights between the treated and the untreated groups except that mice with PC-3 tumours, given MZ-4-71, weighed more than the control animals (Table 1). In experiment I, after 4 weeks of treatment, the volume of DU-145 prostate carcinomas in the group receiving MZ-4-71 was significantly reduced to 30.9 ± 11.1 $mm^3$ compared with the control group (91.2 ± 12.3 $mm^3$), corresponding to a 66% decrease in tumour volume (Figure 1A). After 6 weeks, the final tumour volume and weight were significantly ($P < 0.01$) diminished in animals treated with MZ-4-71 to 37.6 ± 13.5 $mm^3$ and 4.0 ± 1.0 mg, respectively, compared with controls (194.2 ± 55.4 $mm^3$ and 18.6 ± 3.0 mg). Tumour doubling time in mice receiving MZ-4-71 was extended to 56.6 days from 13.4 days calculated for control animals.

In experiment II, therapy with MZ-4-71 also inhibited the growth of PC-3 prostate cancer tumours (Figure 1B). After 4 weeks of therapy, when the experiment was terminated, mean tumour volume and weight were significantly ($P < 0.01$) reduced in animals receiving GH-RH antagonist MZ-4-71 to 119.9 ± 35.3 $mm^3$ and 13.1 ± 3.6 mg compared with those for control animals, which were 397.5 ± 115.6 $mm^3$ and 56.1 ± 8.6 mg respectively. Tumour doubling time was prolonged by MZ-4-71 to 17.8 ± 3 days from 8.7 ± 1.3 days found for control animals ($P < 0.05$).

### Effect of GH-RH antagonist MZ-4-71 on Dunning R-3327 AT-1 tumours in rats

In experiment III, MZ-4-71 effectively suppressed growth of the very fast proliferating Dunning R-3327 AT-1 tumour in rats. After 3 weeks of therapy, tumour volume was 6153 ± 1267 $mm^3$ in the group treated with GH-RH antagonist compared with 11 005 ± 1338 $mm^3$ for control animals ($P < 0.05$) (Figure 2).

The therapy with MZ-4-71 also significantly reduced tumour weight but did not affect body weight. The effects of the treatment on final tumour volume, body and tumour weight and tumour doubling time for rats bearing Dunning R-3327 AT-1 prostate carcinoma are shown in Table 1.

© Cancer Research Campaign 1997

**Figure 1** Tumour volumes in athymic nude mice bearing subcutaneously xenografted DU-145 (**A**) and PC-3 (**B**) human prostate carcinomas during treatment with the growth hormone-releasing hormone antagonist MZ-4-71 administered at a dose of 20 µg twice a day, s.c. Treatment was started when the tumours measured approximately 17–24 mm³ and lasted for 6 and 4 weeks respectively. Vertical bars represent standard error; **P* < 0.05 vs control; ***P* < 0.01 vs control

**Figure 2** Tumour volumes in male Copenhagen rats bearing subcutaneously transplanted rat Dunning R-3327-AT-1 prostate carcinoma during treatment with the GH-RH antagonist MZ-4-71 administered intraperitoneally (i.p.) at a dose of 100 µg per animal per day. Treatment was started immediately after tumour transplantation and lasted for 3 weeks. Vertical bars represent standard error; **P* < 0.05 vs control

### Effect of MZ-4-71 on serum and tissue IGF-I, IGF-II and serum GH levels

The serum levels of GH and IGF-I in control animals and in nude mice treated with the peptide analogue are shown in Table 2. In DU-145 tumour-bearing mice, serum GH levels were significantly reduced in the group treated with MZ-4-71 to 3.61 ± 0.29 ng $ml^{-1}$, compared with control animals (21.27 ± 5.34 ng $ml^{-1}$). The GH-RH antagonist also significantly decreased serum IGF-I levels to 134.5 ± 0.7 ng $ml^{-1}$, compared with 172.5 ± 1 ng $ml^{-1}$ in control animals. Serum levels of IGF-II at the end of the treatment period with MZ-4-71 were similar to those in control animals (Table 2). In animals bearing PC-3 tumours, serum growth hormone was decreased by 46% in the MZ-4-71-treated group (31.1 ± 1.9 ng $ml^{-1}$ vs 57.1 ± 3.6 ng $ml^{-1}$ for control animals; $P < 0.05$). Serum IGF-I was also significantly lower in the animals treated with GH-RH antagonist (93.8 ± 13.6 ng $ml^{-1}$) compared with 135.6 ± 10.6 ng $ml^{-1}$ for control animals, whereas serum IGF-II levels were similar (Table 2). Therapy with MZ-4-71 reduced the IGF-I and IGF-II concentrations in PC-3 tumour tissue below the detection range of the relevant RIA, whereas control tumour tissue showed 145.36 ± 25.22 pg IGF-I 100 $\mu g^{-1}$ protein and 189.11 ± 31.44 pg IGF-II 100 $\mu g^{-1}$ protein (Table 3). After therapy with MZ-4-71, levels of IGF-II in liver tissue of nude mice bearing PC-3 tumours were not significantly changed, while those of IGF-I were reduced from 44.58 ± 4.5 to 32.55 ± 2.5 pg 100 $\mu g^{-1}$ protein ($P < 0.05$)(Table 3). IGF-I and IGF-II measurements in rat serum from the animals implanted with Dunning R-3327AT-1 tumours could not be performed because of an accidental loss of samples.

### Effect of GH-RH antagonist MZ-4-71 on IGF-I receptors of various tumour models

The binding characteristics of receptors for IGF-I in the membranes DU-145, PC-3 and Dunning R-3327-AT-1 tumours were analysed following treatment with GH-RH antagonist MZ-4-71 and the results are presented in Table 4. High-affinity binding sites for IGF were present in the membranes of DU-145

© Cancer Research Campaign 1997

**Table 1** Effect of treatment with GH-RH antagonist MZ-4-71 on tumour volume, body and tumour weight, and tumour doubling time in nude mice bearing xenografts of the human prostate cancer cell lines DU-145 and PC-3 and in rats bearing transplanted Dunning R-3327-AT-1 tumours

| Treatment group | Tumour volume ($mm^3$) | | Body weight (g) | Tumour weight (mg) | Tumour doubling time (days) |
|---|---|---|---|---|---|
| | Initial | Final | | | |
| DU-145 | | | | | |
| Control | 22.3 ± 4.1 | 194.2 ± 35.4 | 34.0 ± 1.0 | 18.6 ± 3 | 13.4 ± 2.6 |
| MZ-4-71 | 24.2 ± 0.8 | 37.6 ± 13.5[a] | 32.0 ± 1.0 | 4.0 ± 1[a] | 56.6 ± 12.2[b] |
| PC-3 | | | | | |
| Control | 17.7 ± 2.8 | 397.5 ± 115.6 | 29.7 ± 0.7 | 56.1 ± 8.6 | 8.7 ± 1.3 |
| MZ-4-71 | 17.8 ± 3.8 | 119.9 ± 35.3[a] | 35.3 ± 0.9[b] | 13.1 ± 3.6[a] | 17.8 ± 3.0[b] |
| Dunning R-3327-AT-1 | | | | | |
| Control | 3 ± 1 | 11005 ± 1388 | 385 ± 17 | 7500 ± 600 | 1.77 ± 0.8 |
| MZ-4-71 | 3 ± 1 | 6153 ± 1267[b] | 392 ± 11 | 5500 ± 900[b] | 1.91 ± 0.3 |

Values are means ± s.e. [a]$P < 0.01$ vs control. [b]$P < 0.05$ vs control.

**Table 2** Serum GH, IGF-I and IGF-II levels in athymic nude mice bearing DU-145 and PC-3 human prostate cancer cell line xenografts after treatment with GH-RH antagonist MZ-4-71

| Treatment group | Growth hormone (GH) (ng $ml^{-1}$) | IGF-I (ng $ml^{-1}$) | IGF-II (ng $ml^{-1}$) |
|---|---|---|---|
| DU-145 | | | |
| Control | 21.27 ± 5.34 | 172.5 ± 1.0 | 25.1 ± 2.9 |
| MZ-4-71 | 3.61 ± 0.29[a] | 134.5 ± 0.7[b] | 21.9 ± 2.2 |
| PC-3 | | | |
| Control | 57.1 ± 3.6 | 135.6 ± 10.6 | 24.8 ± 1.6 |
| MZ-4-71 | 31.1 ± 1.9[a] | 93.8 ± 13.6[b] | 25.6 ± 0.5 |

Values are means ± s.e. [a]$P < 0.01$ vs control. [b]$P < 0.05$ vs control.

tumours ($K_d = 0.82 \pm 0.11$ nM). A significant ($P < 0.01$) increase in binding capacity of IGF-I receptors was observed after treatment with MZ-4-71 ($B_{max} = 244.6 \pm 4.9$ fmol $mg^{-1}$ membrane protein vs 151.4 ± 5.85 fmol $mg^{-1}$ membrane protein for control animals) (Table 4).

In PC-3 tumour membranes, specific high-affinity binding sites for IGF were also demonstrated. In control tumours, the dissociation constant was calculated to be $K_d$ = 0.69 ± 0.25 nM and maximal binding capacity $B_{max} = 90.6 \pm 12.9$ fmol $mg^{-1}$ membrane protein. After therapy with MZ-4-71, $B_{max}$ of the receptors for IGF-I was significantly augmented; i.e. the number of high-affinity receptor sites was increased to 164.4 ± 3.8 fmol $mg^{-1}$ membrane protein (Table 4).

Receptor assays on control rat Dunning R-3327-AT-1 tumours showed high-affinity binding sites for IGF-I ($K_d = 0.49 \pm 0.14$ nM). After 3 weeks of treatment with MZ-4-71, there were no significant changes in the concentration and the affinity of the receptors for IGF-I.

### Effects of IGF-I and GH-RH antagonist MZ-4-71 on cell proliferation in vitro

In order to evaluate the stimulatory effect of IGF-I and a possible anti-proliferative activity of the GH-RH antagonist MZ-4-71 on DU-145 and PC-3 cell lines in vitro, the MTT assay was used. However, IGF-I at concentrations of 5–25 ng $ml^{-1}$ did not significantly stimulate the proliferation of either cell line and the stimulation was calculated to be only between 8% and 23%. At a relatively high concentration of $10^{-5}$ M, GH-RH antagonist MZ-4-71 decreased the proliferation of DU-145 cells by 63.3% compared with the control ($P < 0.05$), but lower concentrations of the antagonist only non-significantly blunted growth of DU-145 cells. The PC-3 prostate cancer cell line was not affected by MZ-4-71, even at concentrations up to $10^{-5}$ M (data not shown).

### DISCUSSION

There are few therapeutic options available for the therapy of androgen-independent prostate carcinoma. Suramin, a polysulphonated naphthylurea, primarily used in the treatment of parasitic disorders, produces a clinical response rate of approximately 50% in patients with advanced, androgen-independent prostate cancers (Eisenberger et al, 1993). The actual mechanism of anti-tumour

**Table 3** Tumour and liver IGF-I and IGF-II levels in athymic nude mice bearing PC-3 xenografts after treatment with the GH-RH antagonist MZ-4-71

| Treatment group | Tumour | | Liver | |
|---|---|---|---|---|
| | IGF-I (pg 100 $\mu g^{-1}$ protein) | IGF-II (pg 100 $\mu g^{-1}$ protein) | IGF-I (pg 100 $\mu g^{-1}$ protein) | IGF-II (pg 100 $\mu g^{-1}$ protein) |
| Control | 145.36 ± 25.22 | 189.11 ± 31.44 | 44.58 ± 4.5 | 33.82 ± 6.1 |
| MZ-4-71 | ND | ND | 32.55 ± 2.57[a] | 28.36 ± 3.1 |

All values are means ± s.e. [a]$P < 0.05$. ND, ≠ not detectable.

© Cancer Research Campaign 1997

Table 4 Binding characteristics of receptors for IGF-I in membranes of DU-145, PC-3 and Dunning R-3327-AT-1 prostate carcinomas after in vivo treatment with GH-RH antagonist MZ-4-71

| Treatment group | $K_d$ (nM) | $B_{max}$ (fmol mg$^{-1}$ membrane protein) |
|---|---|---|
| DU-145 | | |
| Control | 0.82 ± 0.11 | 151.4 ± 5.85 |
| MZ-4-71 | 0.78 ± 0.1 | 244.6 ± 4.9[a] |
| PC-3 | | |
| Control | 0.69 ± 0.25 | 90.6 ± 12.9 |
| MZ-4-71 | 0.87 ± 0.13 | 164.4 ± 3.8[b] |
| Dunning R-3327-AT-1 | | |
| Control | 0.49 ± 0.14 | 71.4 ± 8.9 |
| MZ-4-71 | 0.80 ± 0.08 | 100.3 ± 3.6 |

Binding characteristics were obtained from ten-point displacement experiments. Significance was calculated with Duncan's new multiple range test. All values represent means ± s.e. of 2–3 experiments, each done in triplicate. [a]$P < 0.01$ vs control. [b]$P < 0.05$ vs control.

activity of this drug is unknown, but inhibition of binding of various growth factors to their receptors has been suggested as a possibility. Pollak and Richard (1990) blocked the IGF-I-stimulated proliferation of human osteosarcoma cells with suramin, reinforcing the hypothesis of the possible importance of the GH–IGF-I axis in tumour growth stimulation. Human DU-145 and PC-3 prostate cancer cell lines and rat Dunning R-3327 AT-1 prostate carcinoma are suitable models of the advanced, hormone-insensitive stage of this malignant disease. Our work shows that GH-RH antagonist MZ-4-71 effectively inhibits the growth of DU-145 and PC-3 prostate cancer in nude mice and prolongs tumour doubling time. Our findings on this GH-RH antagonist are in accordance with previous studies on osteosarcoma cell lines (Pinski et al, 1995) and human small-cell and non-small-cell lung carcinomas (Pinski et al, 1996), in which MZ-4-71 effectively inhibited tumour growth.

GH receptors have been identified in a large number of tissues, among them the human prostate and PC-3 prostate cancer cell line (Reiter et al, 1995). Treatment with MZ-4-71 significantly reduced serum GH levels in nude mice bearing DU-145 or PC-3 prostate carcinomas. This suggests that some of the inhibitory effect of the GH-RH antagonist might be attributed to the reduction in serum GH concentration. GH directly regulates the IGF-I synthesis in the liver and other organs (Macaulay, 1992). A significant fall in GH levels, induced by the GH-RH antagonists, could, through mechanisms involving suppression of IGFs, be of major importance for the inhibition of tumour growth. Measurements of serum IGF levels can be affected by the presence of IGF-binding proteins, but our assays were performed after acid–ethanol cryo-precipitation extraction (Breier et al, 1991; Daughaday et al, 1980). Recently, Lee et al (1996) examined acid–ethanol extraction and acid–chromatography procedure for IGF and reported that the results were nearly identical. Only small amounts of residual small molecular weight IGF-binding proteins were found in the acid–ethanol extraction (Lee et al, 1996). Crawford et al (1992) also compared various extraction methods for IGF-I in rat serum, including acid–ethanol extraction with HPLC method. Their results show that IGF-I levels found after acid–ethanol extraction are similar to those obtained by HPLC. Crawford et al (1992) also indicated that the acid–ethanol extraction method of Daughaday et al (1980), originally validated for human serum, is also satisfactory for use with rat serum. Our work shows that, in nude mice treated with the GH-RH antagonist MZ-4-71, serum IGF-I levels were significantly lower compared with control animals. There are conflicting data about the role of the IGF system in the biology of DU-145 and PC-3 human prostate cancer cell lines. Pietrzkowski et al (1993) have reported that DU-145 and PC-3 cells maintain an autocrine production of IGF-I. They showed that the concentration of autocrine IGF-I was sufficient to autophosphorylate the IGF-I receptor and to sustain growth in serum-free cell culture conditions. This autocrine loop, in which produced IGF-I activates its receptors, is at variance with a study by Iwamura et al (1993), who found IGF-I receptors in both cell lines, but could not detect autocrine production of IGF-I. Similarly, Figueroa et al (1995) and Angelloz-Nicoud and Binoux (1995) found an expression of IGF-II and IGF receptors in DU-145 and PC-3 prostate cancer cells respectively, but failed to detect mRNA for IGF-I. Their data suggest an IGF-II-dependent autocrine growth loop.

In our in vitro experiments, addition of 5–25 ng ml$^{-1}$ IGF-I had no stimulatory effect on proliferation in DU-145 and PC-3 cell lines. This is in accord with Pietrzkowski et al (1993), who showed that substantial amounts of IGF-I are secreted by the respective cell lines into the medium and that further exogenous IGF-I had no additional effect on cell proliferation. The determination of GH-RH and GH receptors in prostate cancer and other tumours is the subject of our ongoing investigations. The lack of a dose-dependent effect of GH-RH antagonist MZ-4-71 on the proliferation of DU-145 and PC-3 prostate cancer cells in vitro suggests that no receptors for GH-RH were present on the membranes of these cells or that these cell lines could have undergone changes in GH-RH receptor content during long-term passages in cell cultures. Examples of the loss of peptide receptors after multiple passages are well known (Pinski et al, 1994). Thus, in a study on human colonic and gastric tumour cells, Watson et al (1988) found that the newly established cell lines were stimulated by pentagastrin at passage 2, but long-established cell lines did not respond to pentagastrin, indicating that tumour cell response to hormones was decreased or lost during in vitro cultures. These phenomena might explain the differences between the extent of tumour growth inhibition in vivo and in vitro obtained with our peptides. The doses of MZ-4-71 used in vitro to achieve an inhibition of proliferation of DU-145 tumours were very high. The concentration of MZ-4-71 in the blood of nude mice given a subcutaneous injection of this antagonist at a dose of 20 μg per animal is approximately 20 pM (Pinski et al, 1995), i.e. 500 000 times lower than the dose of MZ-4-71 required to inhibit the growth of DU-145 cells in vitro (10 μM). It would also be intriguing to speculate whether a possible relationship might exist between GH and GH-RH receptors and IGF-I and IGF-II production in tumours, since both these growth factors appear to be involved in PC-3 and DU-145 cell proliferation (Pietrzkowski et al, 1993; Angelloz-Nicoud and Binoux, 1995; Figueroa et al, 1995). However, in the absence of this information, the inhibitory effect of MZ-4-71 on the growth of androgen-independent prostatic cancers in vivo observed in our study may be attributed to the suppression of pituitary GH and hepatic IGF-I secretion and only tentatively to unknown mechanisms leading to an inhibition of autocrine/paracrine IGF-I and IGF-II production and/or lowering of tumour IGF-I and IGF-II levels.

 © Cancer Research Campaign 1997

In our study, tissue concentrations of IGF-I and -II were measured in the liver and tumours of treated and untreated PC-3 tumour-bearing animals. In the liver, IGF-I levels were significantly blunted in the MZ-4-71-treated group, whereas IGF-II levels did not change. On the other hand, in PC-3 tumours, both IGF-I and IGF-II fell below the detection range of the specific RIAs after the treatment with MZ-4-71.

Our findings, which show the presence of high-affinity binding sites for IGF-I in membranes of both PC-3 and DU-145 tumours, are in agreement with results previously reported by other groups (Pietrzkowski et al, 1993; Iwamura et al, 1993; Figueroa et al, 1995). IGF-I and IGF-II bind with different affinities to type 1 IGF receptor, which is thought to mediate the biological effects of both ligands through tyrosine kinase-type activity (Cohen et al, 1994). We observed that chronic treatment of nude mice with the GH-RH antagonists produced an increase of the concentration of IGF-I receptors on both tumours. This phenomenon might be an indication of a compensatory up-regulation of IGF-I receptor synthesis caused by suppression of the endocrine or local production of IGF-I.

Dunning R-3327 AT-1 is a fast growing anaplastic, androgen-independent rat prostate cancer derived from the rat Dunning-H tumour (Isaacs et al, 1978). In this model, the tumour growth was also decreased in the GH-RH antagonist-treated group. The mechanism of action of GH-RH antagonist in this prostate cancer is unclear. It has been demonstrated that Dunning R-3327 tumours express GH receptors (Sinowatz et al, 1991). Thus, the decrease in the levels of GH could contribute to the inhibition of tumours. Tumour growth suppression could also be caused by inhibition of IGF-I levels, since Kovacs et al (1996) showed that chronic administration of MZ-4-71 reduces serum IGF-I concentration in rats by 15%. High-affinity receptors for IGF-I were demonstrated in membranes of Dunning R-3327 AT-1 tumours, but chronic treatment of rats with the GH-RH antagonist did not produce a significant increase of the concentration of IGF-I receptors.

Our study shows that, in androgen-independent prostate cancer models, GH-RH antagonist MZ-4-71 can inhibit tumour growth and reduce serum IGF-I levels. Moreover, tumour tissue IGF-I and -II concentrations were decreased in the human PC-3 and DU-145 cell lines. If similar effects can be achieved in men, GH-RH antagonists might be considered for the development of new approaches to the treatment of patients with prostatic carcinoma who have relapsed following conventional androgen deprivation treatment.

### ABBREVIATIONS

GH, growth hormone; GH-RH, growth hormone-releasing hormone; IGF-I, insulin-like growth factor-I; IGF-II, insulin-like growth factor-II; IGFR, insulin-like growth factor receptor; EGF, epidermal growth factor; LH-RH, luteinizing hormone-releasing hormone; TGF-α and -β, transforming growth factor-α and -β; DMSO: dimethyl sulphoxide.

### ACKNOWLEDGEMENTS

The authors thank Ms Dora Rigo, Ms Elena Glotser and Mr Harold Valerio for technical assistance and Professor J Frick for his support. The gifts of materials used in RIA from the National Hormone and Pituitary Program (NHPP) of the National Institute of Diabetes and Digestive and Kidney Diseases (NATCH) and from Dr AF Parlow (Pituitary Hormones and Antisera Center, Torrance, CA, USA) are greatly appreciated. The work described in this paper was supported by the Medical Research Service of the Veterans Affairs Department (to AVS) and by a grant from Pierre Fabre Medicament to Tulane University School of Medicine and. AJ is a recipient of a fellowship from the Fond zur Förderung der wissenschaftlichen Forschung, Austria.

### REFERENCES

Angelloz-Nicoud P and Binoux M (1995) Autocrine regulation of cell proliferation by the insulin-like growth factor (IGF) and IGF binding protein-3 protease system in a human prostate carcinoma cell line (PC-3). *Endocrinology* **136**: 5485–5492

Breier BH, Gallaher BW and Gluckman PD (1991) Radioimmunoassay for insulin-like growth factor-I: solutions to some potential problems and pitfalls. *J Endocrinol* **128**: 347–357

Cohen P, Peehl DM and Rosenfeld RG (1994) The IGF axis in the prostate. *Horm Metab Res* **26**: 81–84

Crawford BA, Martin JI, Howe CJ, Handelsman DJ and Baxter RC (1992) Comparison of extraction methods for insulin-like growth factor-I in rat serum. *J Endocrinol* **134**: 169–176

Daughaday WH, Mariz IK and Blethen SL (1980) Inhibition of access of bound somatomedin to membrane receptor and immunobinding sites: a comparison of radioreceptor and radioimmunoassay of somatomedin in native and acid–ethanol extracted serum. *J Clin Endocrinol Metab* **51**: 781–788

Davies P and Eaton CL (1991) Regulation of prostate growth. *J Endocrinol* **131**: 5–17

D'Ercole AJ, Stiles AD and Underwood LE (1984) Tissue concentrations of somatomedin C: further evidence for multiple sites of synthesis and paracrine or autocrine mechanisms of action. *Proc Natl Acad Sci USA* **81**: 935–939

Eisenberger MA, Reyno LM, Jodrell DI, Sinibaldi VJ, Tkaczuk KH, Sridhara R, Zuhowski EG, Lowitt MH, Jacobs SC and Egorin MJ (1993) Suramin, an active drug for prostate cancer: interim observations in a phase I trial. *J Natl Cancer Inst* **85**: 611–621

Figueroa JA, Lee AV, Jackson JG and Yee D (1995) Proliferation of cultured human prostate cancer cells is inhibited by insulin-like growth factor (IGF) binding protein-1: evidence for an IGF-II autocrine growth loop. *J Clin Endocrinol Metab* **80**: 3476–3482

Froesch ER, Schmid C, Schwander J and Zapf J (1985) Actions of insulin-like growth factors. *Annu Rev Physiol* **47**: 443–467

Garnick MB (1993) Prostate cancer: screening, diagnosis, and management. *Ann Int Med* **118**: 804–818

Isaacs JT, Heston WD, Weissman R and Coffey DS (1978) Animal models for the hormone-sensitive and -insensitive prostatic adenocarcinomas, Dunning R-3327-H, R-3327-HI, and R-3327-AT. *Cancer Res* **38**: 4353–4359

Iwamura M, Sluss PM, Casamento JB and Cockett ATK (1993) Insulin-like growth factor I: action and receptor characterization in human prostate cancer cell lines. *Prostate* **22**: 243–252

Janik P, Briand P and Hartmann NR (1975) The effect of estrone–progesterone treatment on cell proliferation kinetics of hormone-dependent GR mouse mammary tumors. *Cancer Res* **35**: 3698–370

Kaighn ME, Shankar Narayan K, Ohnuki Y, Lechner JF and Jones LW (1979) Establishment and characterization of a human prostatic carcinoma cell line (PC-3). *Invest Urol* **17**: 16–23

Kovács M, Zarándi M, Halmos G, Groot K and Schally AV (1996) Effects of acute and chronic administration of a new potent antagonist of growth hormone-releasing hormone in rats: mechanisms of action. *Endocrinology* **137**: 5364–5369

Lee PDK, Baker BK, Liu F, Kwan EYW and Hintz RL (1996) A homologous radioimmunoassay for rat insulin-like growth factor (IGF-I). Implications for studies of human IGF-I physiology. *J Clin Endocrinol Metab* **81**: 2002–2005

Lu-Yao GL, Potosky AL, Albertsen PC, Wasson JH, Barry MJ and Wennberg JE (1996) Follow-up prostate cancer treatments after radical prostatectomy: a population-based study. *J Natl Cancer Inst* **88**: 166–173

Macaulay VM (1992) Insulin-like growth factors and cancer. *Br J Cancer* **65**: 311–320

Munson JP and Rodbard D (1980) A versatile computerized approach for characterization of ligand binding systems. *Anal Biochem* **107**: 220–239

Parker SL, Tong T, Bolden S and Wingo PA (1996) Cancer statistics, 1996. *CA Cancer J Clin* **65**: 5–27

Pietrzkowski Z, Mulholland G, Gomella L, Jameson BA, Wernicke D and Baserga R (1993) Inhibition of growth of prostatic cancer cell lines by peptide analogues of insulin-like growth factor 1. *Cancer Res* **53**: 1102–1106

© Cancer Research Campaign 1997

Pinski J, Schally AV, Groot K, Halmos G, Szepeshazi K, Zarandi M and Armatis P (1995) Inhibition of growth of human osteosarcomas by antagonists of growth hormone-releasing hormone. *J Natl Cancer Inst* **87**: 1787–1794

Pinski J, Schally AV, Halmos G, Szepeshazi K and Groot K (1994) Somatostatin analogues and bombesin/gastrin-releasing peptide antagonist RC-3095 inhibit the growth of human glioblastomas in vitro and in vivo. *Cancer Res* **54**: 5895–5901

Pinski J, Schally AV, Jungwirth A, Groot K, Halmos G, Armatis P, Zarandi M and Vadillo-Buenfil M (1996) Inhibition of growth of human small-cell and non-small-cell lung carcinomas by antagonists of growth hormone-releasing hormone (GH-RH). *Int J Oncol* **9**: 1099–1105

Plumb JA, Milroy R and Kaye SB (1989) Effects of the pH dependence of 3-(4,5-dimethylthiazol-2-yl)-2,5,-diphenyl-tetrazolium bromide-formazan absorption on chemosensitivity determined by a novel tetrazolium-based assay. *Cancer Res* **49**: 4435–4440

Pollak M and Richard M (1990) Suramin blockade of insulin-like growth factor I-stimulated proliferation of human osteosarcoma cells. *J Natl Cancer Inst* **82**: 1349–1352

Reiter E, Kecha O, Hennuy B, Lardinois S, Klug M, Bruyninx M, Closset J and Hennen G (1995) Growth hormone directly affects the function of the different lobes of the rat prostate. *Endocrinology* **136**: 3338–3345

Reubi JC, Waser B, Schaer JC and Markwalder R (1995) Somatostatin receptors in human prostate and prostate cancer. *J Clin Endocrinol Metabol* **80**: 2806–2814

Schally AV, Comaru-Schally AM and Gonzales-Barcena D (1990) Present status of agonistic and antagonistic analogs of LH-RH in the treatment of advanced prostate cancer. *Biomed Pharmacother* **46**: 465–471

Sharifi R, Soloway M and the Leuprolide Study Group (1990) Clinical study of leuprolide depot formulation in the treatment of advanced prostate cancer. *J Urol* **143**: 68–71

Sinowatz F, Breipohl W, Waters MI, Lincoln D, Lobie PE and Amselgruber W (1991) Growth hormone receptor expression in the Dunning R 3327 prostatic carcinoma of the rat. *Prostate* **19**: 273–278

Steel RGD and Torrie J (1976) *Principles and Procedures of Statistics*, p. 114. McGraw-Hill: New York

Stone KR, Mickey DD, Wunderli H, Mickey GH and Paulson DF (1978) Isolation of a human prostate carcinoma cell line (DU-145). *Int J Cancer* **21**: 274–281

Tanaka H, Asami O, Hayano T, Sasaki I, Yoshitake Y and Nishikawa K (1989) Identification of a family of insulin-like growth factor II secreted by cultured rat epithelial-like cell line 18,54-SF: application of a monoclonal antibody. *Endocrinology* **124**: 870–877

Walsh PC, Partin AW and Epstein JI (1994) Cancer control and quality of life following anatomical radical retropubic prostatectomy: results at 10 years. *J Urol* **152**: 1831–1836

Watson SA, Durrant L and Morris DL (1988) Growth promoting action of gastrin on human colonic and gastric tumor cell cultures in vitro. *Br J Surg* **75**: 342–345

Zarandi M, Horvath JE, Halmos G, Pinski J, Nagy A, Groot K and Schally AV (1994) Synthesis and biological activities of highly potent antagonist of growth hormone-releasing hormone. *Proc Natl Acad Sci USA* **91**: 12298–12302

*© Cancer Research Campaign 1997*


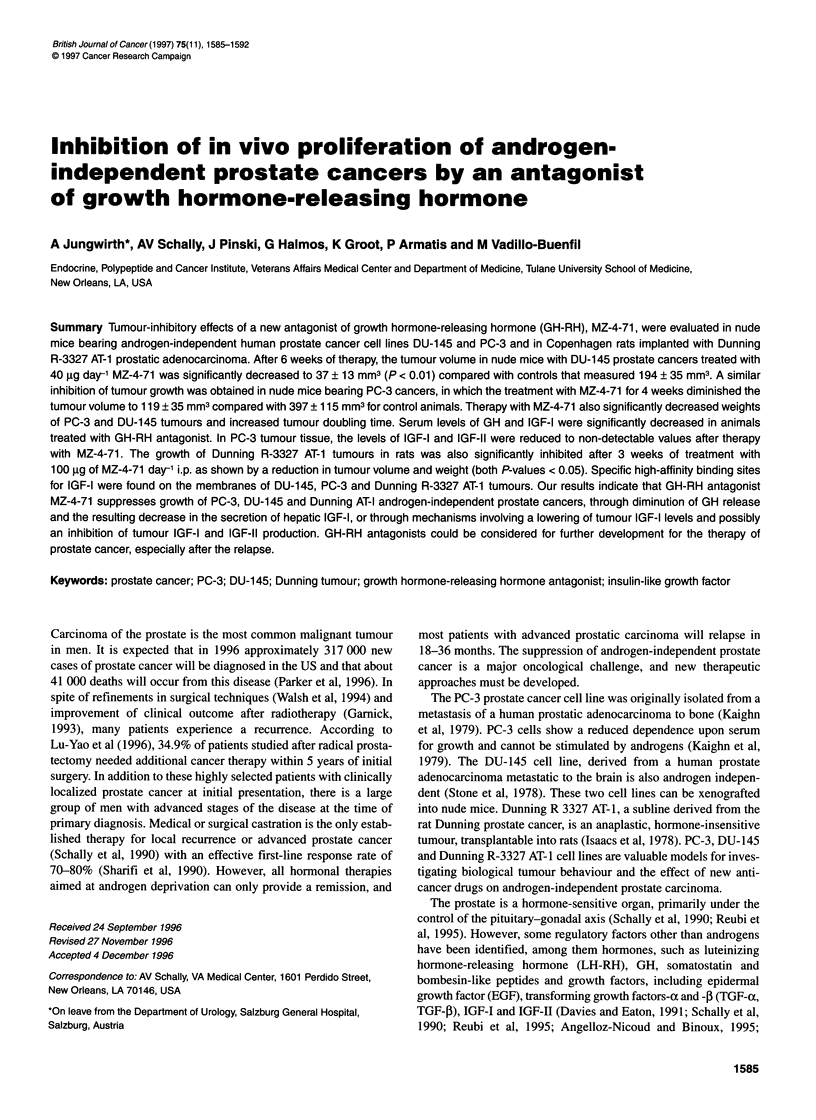

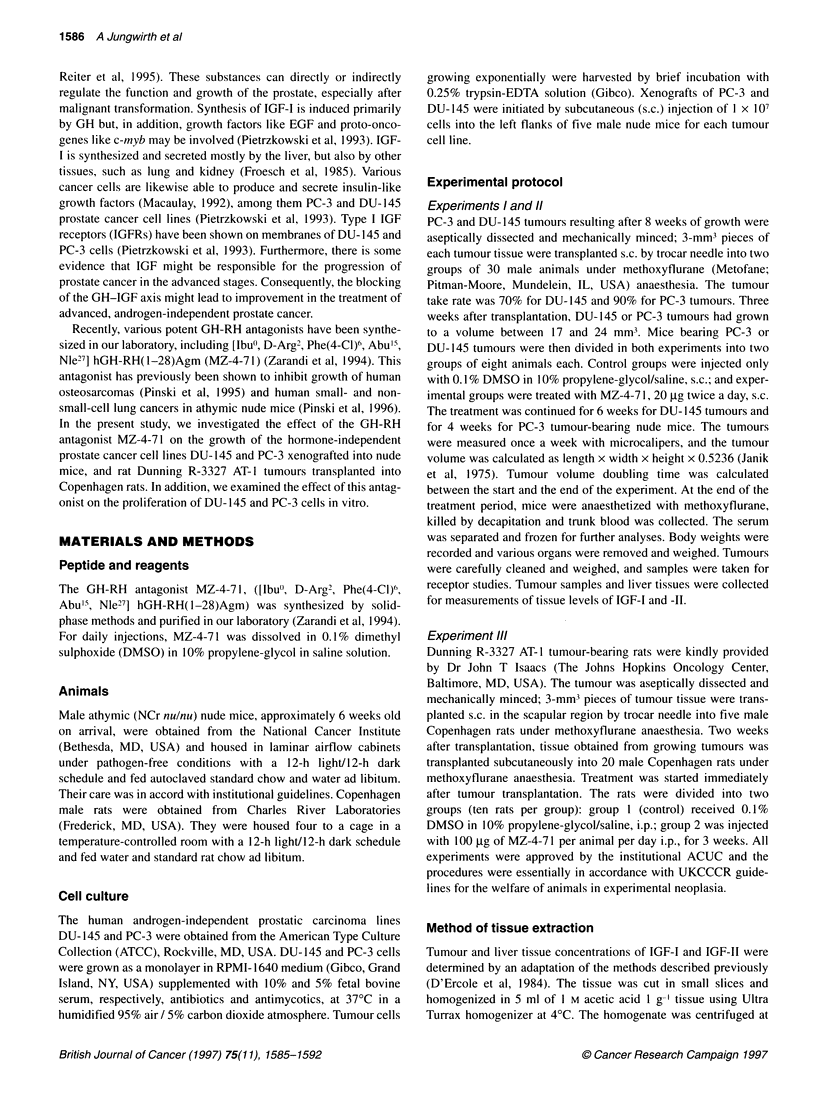

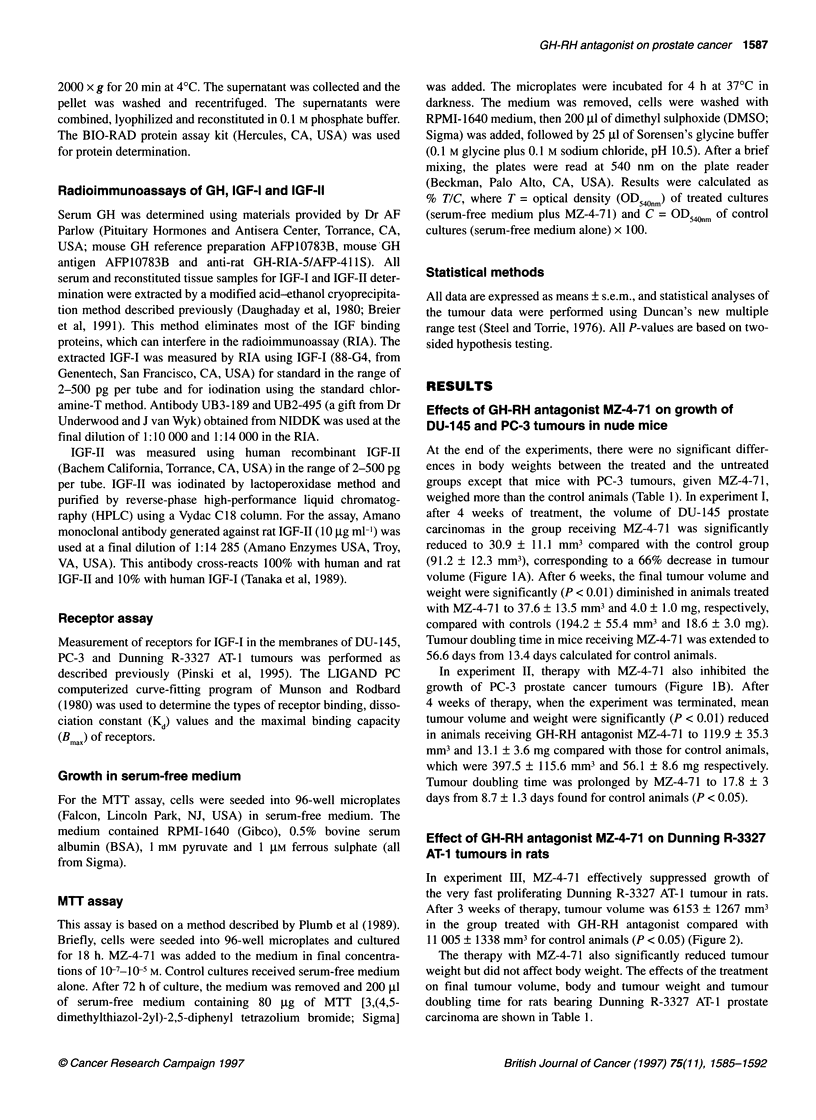

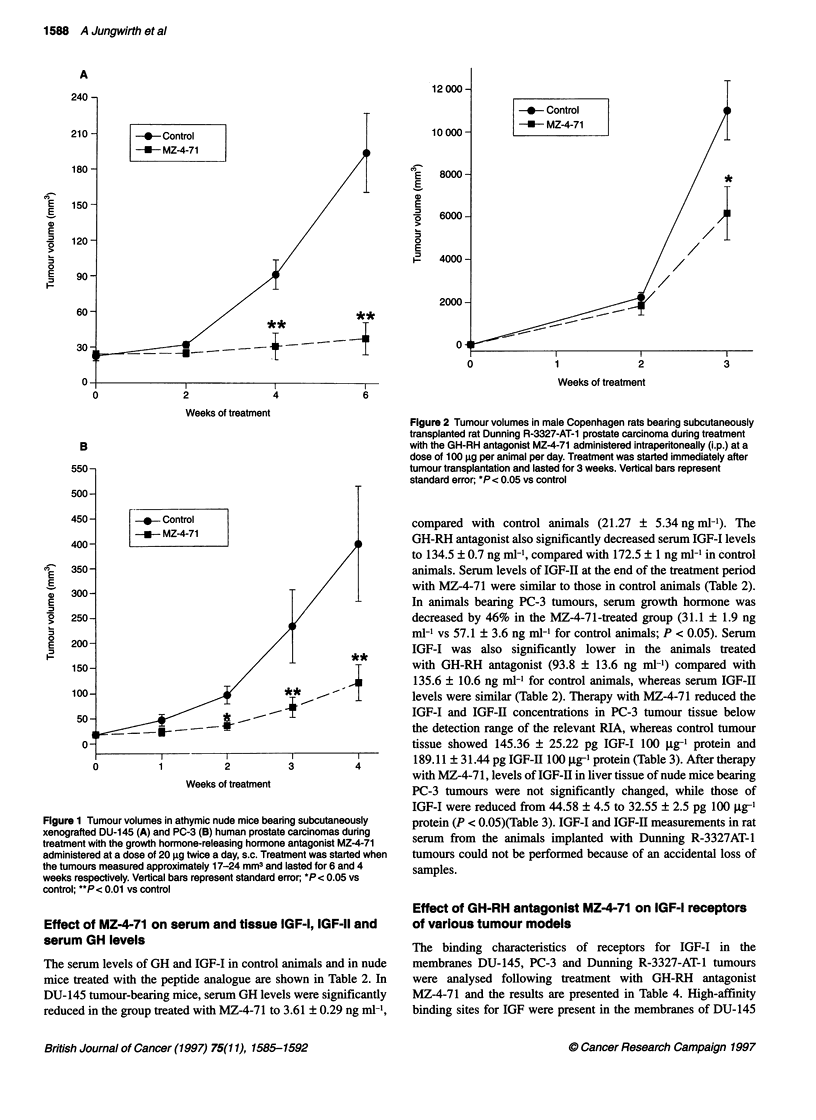

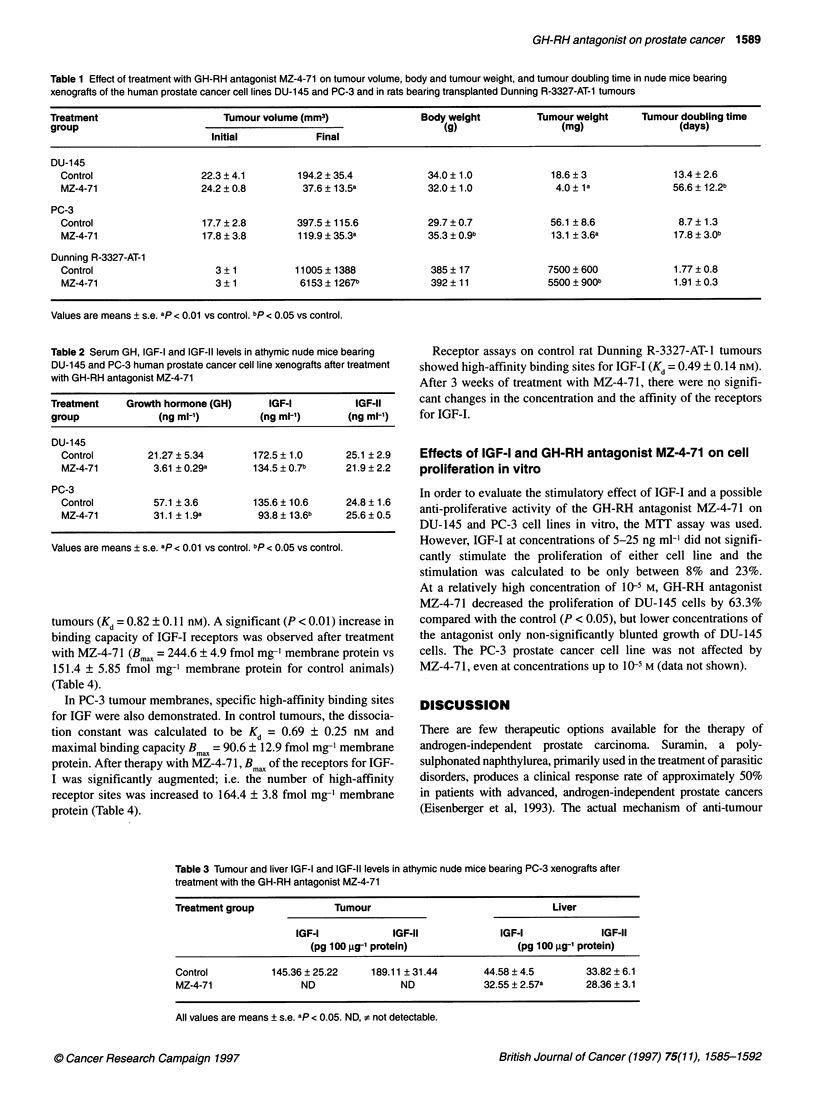

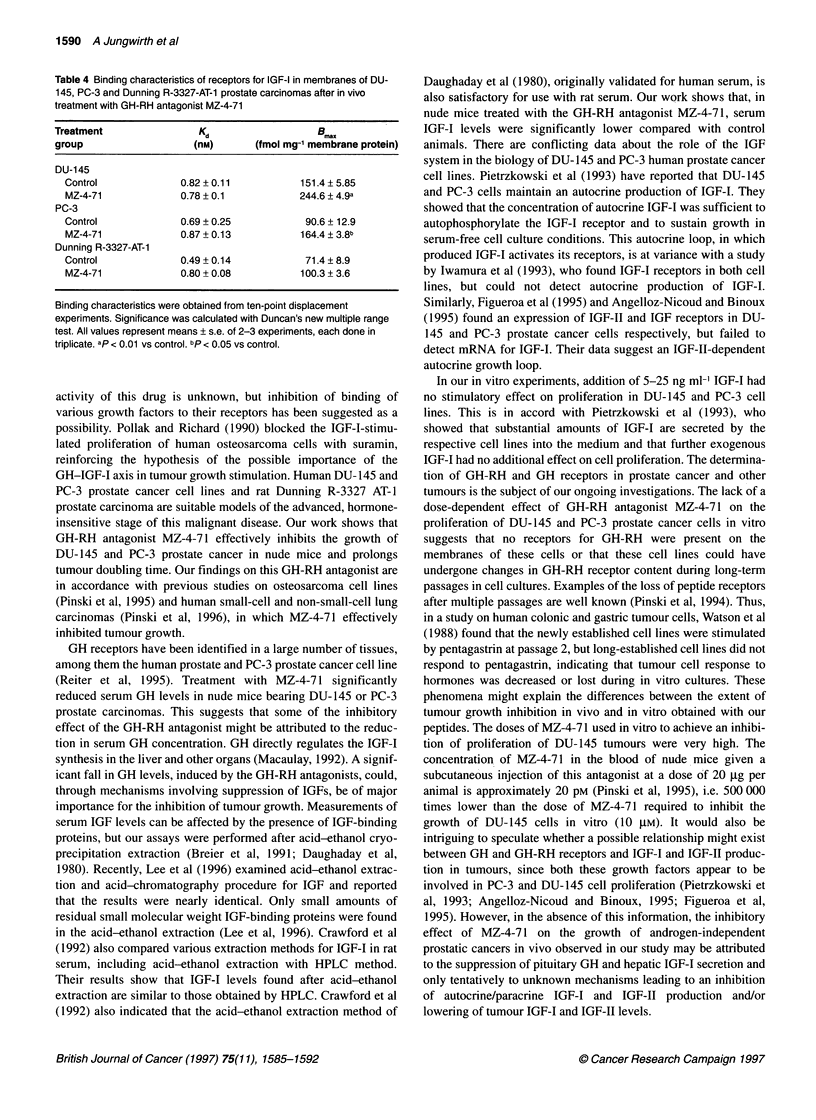

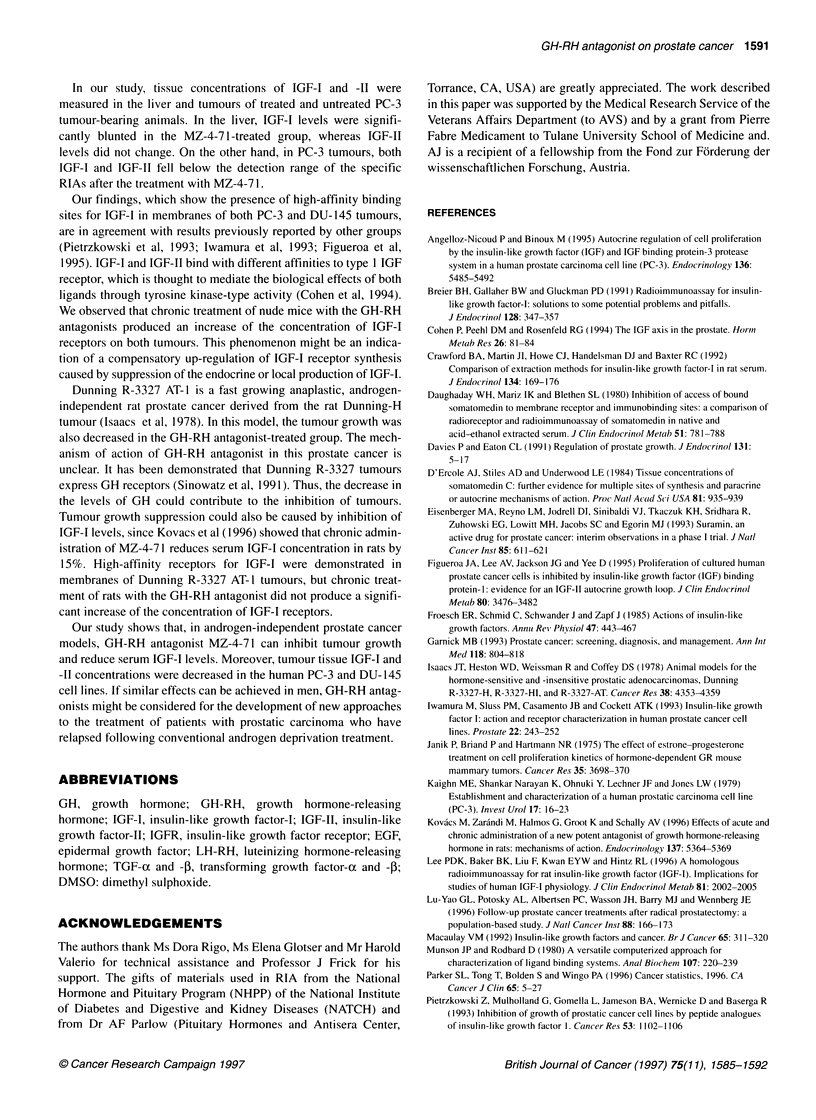

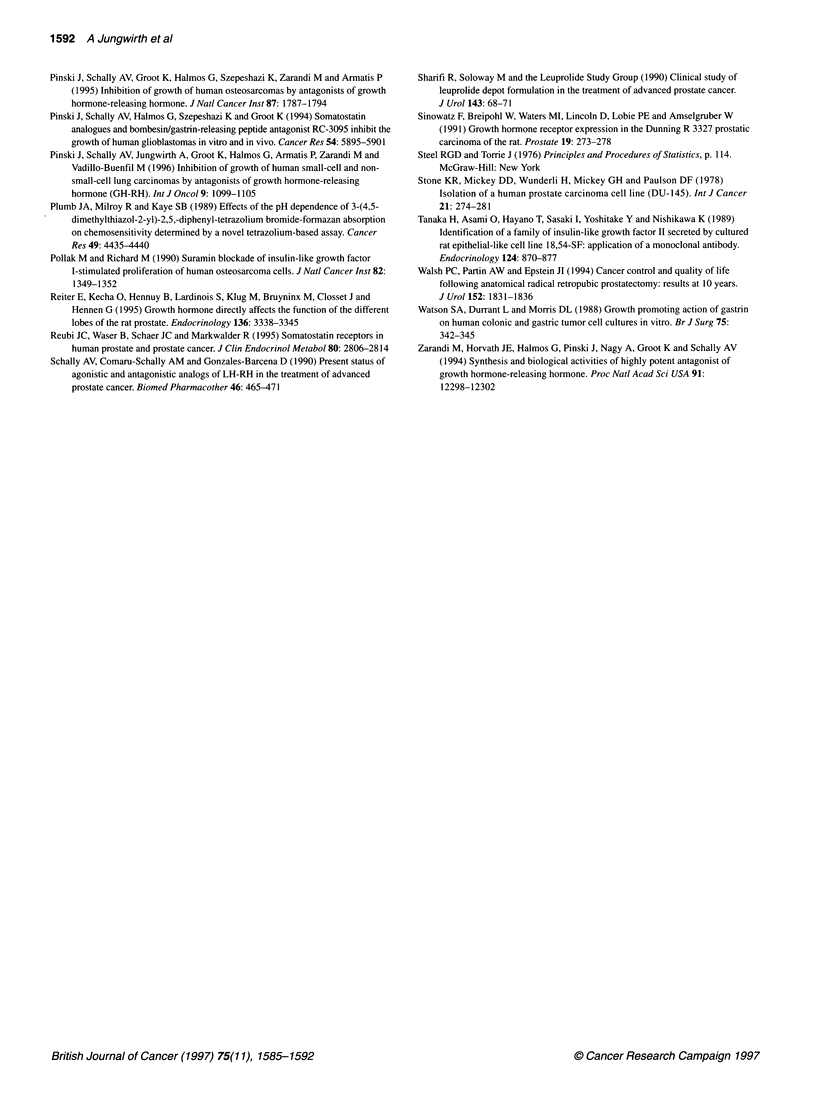


## References

[OCR_00844] Angelloz-Nicoud P., Binoux M. (1995). Autocrine regulation of cell proliferation by the insulin-like growth factor (IGF) and IGF binding protein-3 protease system in a human prostate carcinoma cell line (PC-3).. Endocrinology.

[OCR_00850] Breier B. H., Gallaher B. W., Gluckman P. D. (1991). Radioimmunoassay for insulin-like growth factor-I: solutions to some potential problems and pitfalls.. J Endocrinol.

[OCR_00855] Cohen P., Peehl D. M., Rosenfeld R. G. (1994). The IGF axis in the prostate.. Horm Metab Res.

[OCR_00875] D'Ercole A. J., Stiles A. D., Underwood L. E. (1984). Tissue concentrations of somatomedin C: further evidence for multiple sites of synthesis and paracrine or autocrine mechanisms of action.. Proc Natl Acad Sci U S A.

[OCR_00864] Daughaday W. H., Mariz I. K., Blethen S. L. (1980). Inhibition of access of bound somatomedin to membrane receptor and immunobinding sites: a comparison of radioreceptor and radioimmunoassay of somatomedin in native and acid-ethanol-extracted serum.. J Clin Endocrinol Metab.

[OCR_00871] Davies P., Eaton C. L. (1991). Regulation of prostate growth.. J Endocrinol.

[OCR_00880] Eisenberger M. A., Reyno L. M., Jodrell D. I., Sinibaldi V. J., Tkaczuk K. H., Sridhara R., Zuhowski E. G., Lowitt M. H., Jacobs S. C., Egorin M. J. (1993). Suramin, an active drug for prostate cancer: interim observations in a phase I trial.. J Natl Cancer Inst.

[OCR_00887] Figueroa J. A., Lee A. V., Jackson J. G., Yee D. (1995). Proliferation of cultured human prostate cancer cells is inhibited by insulin-like growth factor (IGF) binding protein-1: evidence for an IGF-II autocrine growth loop.. J Clin Endocrinol Metab.

[OCR_00893] Froesch E. R., Schmid C., Schwander J., Zapf J. (1985). Actions of insulin-like growth factors.. Annu Rev Physiol.

[OCR_00897] Garnick M. B. (1993). Prostate cancer: screening, diagnosis, and management.. Ann Intern Med.

[OCR_00901] Isaacs J. T., Heston W. D., Weissman R. M., Coffey D. S. (1978). Animal models of the hormone-sensitive and -insensitive prostatic adenocarcinomas, Dunning R-3327-H, R-3327-HI, and R-3327-AT.. Cancer Res.

[OCR_00906] Iwamura M., Sluss P. M., Casamento J. B., Cockett A. T. (1993). Insulin-like growth factor I: action and receptor characterization in human prostate cancer cell lines.. Prostate.

[OCR_00911] Janik P., Briand P., Hartmann N. R. (1975). The effect of estrone-progesterone treatment on cell proliferation kinetics of hormone-dependent GR mouse mammary tumors.. Cancer Res.

[OCR_00916] Kaighn M. E., Narayan K. S., Ohnuki Y., Lechner J. F., Jones L. W. (1979). Establishment and characterization of a human prostatic carcinoma cell line (PC-3).. Invest Urol.

[OCR_00926] Lee P. D., Baker B. K., Liu F., Kwan E. Y., Hintz R. L. (1996). A homologous radioimmunoassay for rat insulin-like growth factor-I (IGF-I): implications for studies of human IGF-I physiology.. J Clin Endocrinol Metab.

[OCR_00930] Lu-Yao G. L., Potosky A. L., Albertsen P. C., Wasson J. H., Barry M. J., Wennberg J. E. (1996). Follow-up prostate cancer treatments after radical prostatectomy: a population-based study.. J Natl Cancer Inst.

[OCR_00935] Macaulay V. M. (1992). Insulin-like growth factors and cancer.. Br J Cancer.

[OCR_00936] Munson P. J., Rodbard D. (1980). Ligand: a versatile computerized approach for characterization of ligand-binding systems.. Anal Biochem.

[OCR_00939] Parker S. L., Tong T., Bolden S., Wingo P. A. (1996). Cancer statistics, 1996.. CA Cancer J Clin.

[OCR_00952] Pinski J., Schally A. V., Groot K., Halmos G., Szepeshazi K., Zarandi M., Armatis P. (1995). Inhibition of growth of human osteosarcomas by antagonists of growth hormone-releasing hormone.. J Natl Cancer Inst.

[OCR_00957] Pinski J., Schally A. V., Halmos G., Szepeshazi K., Groot K. (1994). Somatostatin analogues and bombesin/gastrin-releasing peptide antagonist RC-3095 inhibit the growth of human glioblastomas in vitro and in vivo.. Cancer Res.

[OCR_00967] Plumb J. A., Milroy R., Kaye S. B. (1989). Effects of the pH dependence of 3-(4,5-dimethylthiazol-2-yl)-2,5-diphenyl-tetrazolium bromide-formazan absorption on chemosensitivity determined by a novel tetrazolium-based assay.. Cancer Res.

[OCR_00973] Pollak M., Richard M. (1990). Suramin blockade of insulinlike growth factor I-stimulated proliferation of human osteosarcoma cells.. J Natl Cancer Inst.

[OCR_00978] Reiter E., Kecha O., Hennuy B., Lardinois S., Klug M., Bruyninx M., Closset J., Hennen G. (1995). Growth hormone directly affects the function of the different lobes of the rat prostate.. Endocrinology.

[OCR_00983] Reubi J. C., Waser B., Schaer J. C., Markwalder R. (1995). Somatostatin receptors in human prostate and prostate cancer.. J Clin Endocrinol Metab.

[OCR_00986] Schally A. V., Comaru-Schally A. M., Gonzalez-Barcena D. (1992). Present status of agonistic and antagonistic analogs of LH-RH in the treatment of advanced prostate cancer.. Biomed Pharmacother.

[OCR_00991] Sharifi R., Soloway M. (1990). Clinical study of leuprolide depot formulation in the treatment of advanced prostate cancer. The Leuprolide Study Group.. J Urol.

[OCR_00996] Sinowatz F., Breipohl W., Waters M. I., Lincoln D., Lobie P. E., Amselgruber W. (1991). Growth hormone receptor expression in the Dunning R 3327 prostatic carcinoma of the rat.. Prostate.

[OCR_01005] Stone K. R., Mickey D. D., Wunderli H., Mickey G. H., Paulson D. F. (1978). Isolation of a human prostate carcinoma cell line (DU 145).. Int J Cancer.

[OCR_01010] Tanaka H., Asami O., Hayano T., Sasaki I., Yoshitake Y., Nishikawa K. (1989). Identification of a family of insulin-like growth factor II secreted by cultured rat epithelial-like cell line 18,54-SF: application of a monoclonal antibody.. Endocrinology.

[OCR_01016] Walsh P. C., Partin A. W., Epstein J. I. (1994). Cancer control and quality of life following anatomical radical retropubic prostatectomy: results at 10 years.. J Urol.

[OCR_01021] Watson S. A., Durrant L. G., Morris D. L. (1988). Growth-promoting action of gastrin on human colonic and gastric tumour cells cultured in vitro.. Br J Surg.

[OCR_01026] Zarandi M., Horvath J. E., Halmos G., Pinski J., Nagy A., Groot K., Rekasi Z., Schally A. V. (1994). Synthesis and biological activities of highly potent antagonists of growth hormone-releasing hormone.. Proc Natl Acad Sci U S A.

